# Both overexpression and suppression of an *Oryza sativa* NB-LRR-like gene *OsLSR* result in autoactivation of immune response and thiamine accumulation

**DOI:** 10.1038/srep24079

**Published:** 2016-04-07

**Authors:** Liangchao Wang, Ye, Huachun Liu, Xuejiao Liu, Chuchu Wei, Yuqing Huang, Yujun Liu, Jumin Tu

**Affiliations:** 1 Institute of Crop Science, Zhejiang University, Yu-Hang-Tang Road No 866, Hangzhou, 310058, China

## Abstract

Tight and accurate regulation of immunity and thiamine biosynthesis is critical for proper defence mechanisms and several primary metabolic cycles in plants. Although thiamine is known to enhance plant defence by priming, the mechanism by which thiamine biosynthesis responds to immune signals remains poorly understood. Here we identified a novel rice (*Oryza sativa* L.) NB-LRR gene via an insertion mutation, this mutant confesses a low seed setting phenotype and the corresponding genetic locus was named *OsLSR* (*Low seed setting related*). Comparing with wildtype plant, both overexpression and suppression of *OsLSR* lead to the autoactivation of the rice immune system and accumulation of thiamine, which result in a great fitness cost and yield penalty. Moreover, when fused with eGFP at their C terminus, two fragments, OsLSR1-178 and OsLSR464-546, localized to chloroplasts where thiamine is produced. Our result suggests that *OsLSR* differs from traditional NB-LRR genes. Its expression is closely related to the immune status and thiamine level in plant cells and should be maintained within a narrow range for rice growth.

Plants continually face attempted pathogen invasions and, in response, they use multiple mechanisms to fight against pathogen attacks. The resistance (*R*) gene-mediated defence response is one of these strategies. Most plant *R* genes belong to the nucleotide-binding leucine-rich repeat (NB-LRR) gene family.

A typical NB-LRR protein contains a C-terminal LRR domain, a central NB domain, and a variable N-terminal domain, which usually exhibits either a Toll/Interleukin-1 receptor (TIR) domain or a coiled-coil (CC) domain^1^. Most NB-LRR proteins function as intracellular immune receptors and are expressed at a constant basal level in plant cells. They detect pathogen-derived effectors either directly or indirectly and then activate immune responses, including the accumulation of the defence hormones salicylic acid (SA) or jasmonic acid (JA), the production of reactive oxygen species (ROS), and the secretion of numerous PR (pathogenesis-related) proteins. The activation of these various responses eventually leads to localized killing of infected cells and restricted pathogen spread^2^,^3^.

NB-LRR proteins can activate the defence response in multiple subcellular compartments. Arabidopsis RPM1 is located at the plasma membrane, where it activates and triggers defence responses^4^,^5^. Some NB-LRR proteins need to be relocated to the nucleus to trigger full resistance during activation, for example, barley MLA10 and tobacco N^6^,^7^. The chloroplast plays an important role in defence signalling because it is involved in the generation of defence signalling molecules, such as ROS, JA and SA^8^. While some NB-LRR proteins were predicted to be chloroplastic^9^, to date, the chloroplast localization of a NB-LRR protein lacks experimental evidence.

The chloroplast is also where thiamine (vitamin B1) is created^10^. Thiamine and its phosphorylated forms play a fundamental role as an enzymatic cofactor in plant metabolism, including in the Calvin Benson cycle (C3), the pentose phosphate pathway (PPP), and the tricarboxylic acid cycle (TCA). Furthermore, thiamine is well known to trigger the immune response in plants^11^. Thiamine enhances plant disease resistance by priming, leading to a rapid counter attack against pathogen invasion and perturbation of disease progression^12^. Several studies have further shown that thiamine’s ability to enhance or trigger disease resistance is SA-dependent, as this induction or priming fails in mutants that do not accumulate SA^12^,^13^,^14^. Thiamine is de novo synthesized in plants and bacteria. In plants, thiamine is biosynthesized through the separate formation of HMP-PP (4-amino-2-methyl-5-hydroxymethylpyrimidine diphosphate) and HET-P (4-methyl-5-b-hydroxyethylthiazole phosphate); these two molecules then couple to form thiamine monophosphate (TMP) that is subsequently phosphorylated to form thiamine triphosphate (TPP), which is the active form of vitamin B1^10^. Plants encode three enzymes to catalyse these committed steps: HET-P synthase (THI4), HMP-P synthase (THIC) and a bifunctional enzyme HMPPK (HMP-P kinase). HET-P biosynthesis is similar to that in yeast, in which HET-P synthase (THI4p) catalyses the formation of the thiazole moiety from NAD^+^, glycine, and a sulphur from a backbone cysteine within itself^15^. Homologs of plant *THI4* (*THI1*) have been cloned from *maize*^16^, *Alnusglutinosa*^17^, *Arabidopsis thaliana*^18^, and *Oryza sativa*^19^. The HMP-P synthase THIC converts aminoimidazole ribonucleotide (AIR) to hydroxymethylpyrimidine phosphate (HMP-P). *THICs* have been characterized in *Arabidopsis*^20^ and tomato^21^. HMP-P is further phosphorylated to HMP-PP by HMPPK, and the latter catalyses the condensation of HET-P and HMP-PP to form TMP. All these processes occur in the chloroplast^22^. *HMPPKs* have been characterized in Arabidopsis as *TH1*^23^, *Brassica napus* (*BTH1*)^24^, and *Z.mays* (*THI3*)^25^. TMP is dephosphorylated to thiamine, which is then phosphorylated by thiamine phosphorylase (TPKs) to become the active form TPP. Arabidopsis encodes two thiamine phosphorylases, TPK1 and TPK2^26^.

Thiamine biosynthesis is tightly and negatively regulated by TPP through a riboswitch in the *THIC* mRNA^27^. A high level of TPP in the cell leads to an increased level of unstable *THIC* mRNA in which the second intron in the 3′UTR is spliced out. When the TPP level is low, this intron is retained and results in enhanced stability of the mRNA, which increases the translation of THIC protein and consequently increases thiamine biosynthesis. A recent study showed that overexpression of the enzyme transketolase (TK) involved in the C3 cycle provides precursors for producing thiamine, leading to thiamine auxotrophy in tobacco^28^. This result suggests that the thiamine level in a plant cell can also be affected by a basic metabolic cycle. Thiamine biosynthesis is activated during a plant’s response to abiotic and biotic stresses, including oxidative, salt and osmotic stress^29^,^30^, and also by pathogen inoculation^19^. A recent study found that thiamine biosynthesis in response to abiotic stress is mediated by ABA at an early stage^31^. However, the mechanism by which thiamine biosynthesis responds to immune signals remains to be elucidated.

In our current study, we characterized the NB-LRR gene *OsLSR* (Gene Bank Accession No. Os10g0183000), both of its up- and down- regulation triggering immune response of rice plants. These plants constitutively express *PR* genes, accumulate thiamine and showed a weakness. RT-qPCR analysis of thiamine biosynthesis related genes revealed that *OsLSR* overexpression lines accumulate thiamine by enhanced expression of *OsDR8* (Defence-response protein 8), while RNA silence of *OsLSR* leads to a boost in *OsTHIC* transcripts. Our results broaden our understanding of the function of the NB-LRR protein and suggest a possible link between the NB-LRR-mediated immune response and thiamine accumulation, which could be used in rice improvement.

## Results

### *OsLSR* encodes a CC-NB-LRR protein

**Figure 1:**
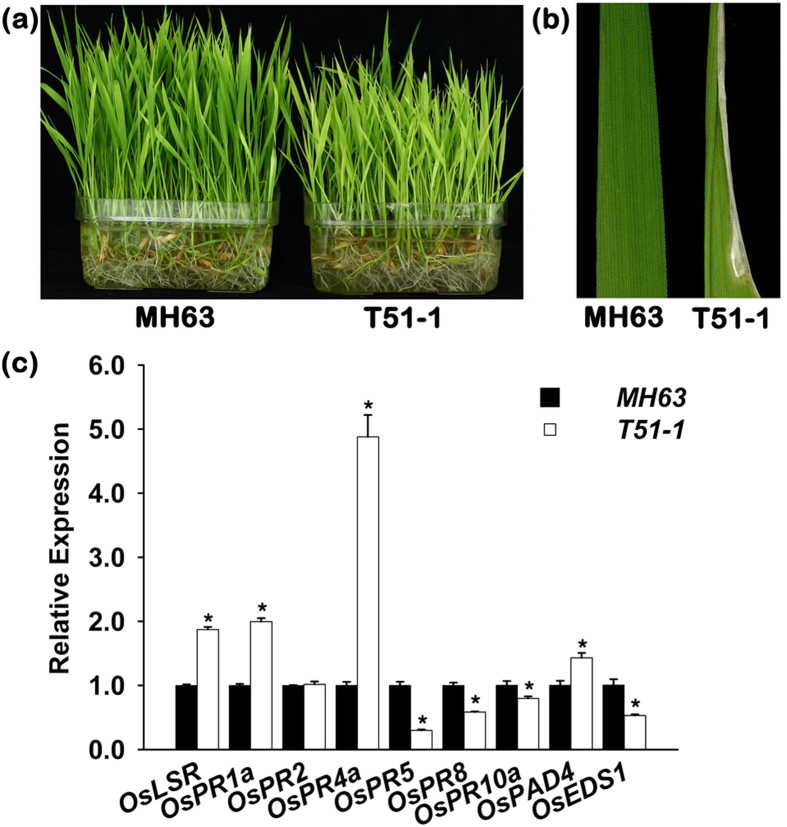
Up regulation of *OsLSR* and its effects on rice morphology and activation of the immune response. (**a**) Yellowish leaf phenotype of T51-1 compared with MH63. (**b**) Leaf tip necrosis in T51-1. (**c**) Transcript levels of *OsLSR* and disease-resistant marker genes in MH63 and T51-1. For the expression analysis, *OsUBQ1* was used as the internal control. Scale bars indicate the means ± SE (n = 3), *P < 0.05(ANOVA) comparing with T51-1 and MH63.

*OsLSR* (*Low Seed setting Related*) was initially cloned as genomic sequences flanking the transgene insertion site in the *Bt* T51-1 line, which has an MH63 background^32^,^33^. The exogenous *Bt* gene was inserted within its promoter region at a distance of 797 bp upstream of the start codon ATG. The T51-1 line showed a yellowish colour with a necrotic tip at the seeding stage (Fig. 1a,b). T51-1 also showed weakness in seed setting and was 10–20% lower than the wild type controls (Supplementary Table S1).

A protein sequence analysis revealed that *OsLSR* encodes a putative disease resistance-like protein (R protein) that has an N-terminal CC domain (1–128aa), a C-terminal LRR domain (458–894aa), and a central NB-ARC domain (165–457aa) (a nucleotide-binding domain shared by APAF-1, certain R gene products and CED-4) (http://www.ebi.ac.uk/interpro/).

### Up-regulation of *OsLSR* triggers the rice immune response

To determine the effect of the insertion, the *OsLSR* transcripts were analysed by RT-qPCR in T51-1 leaf tissue. *OsUBQ1* was used as an internal control, and the analysis showed a slight increase of *OsLSR* in T51-1 compared to MH63 (Fig. 1c). Thus, *OsLSR* is up-regulated in T51-1.

The up-regulation of NB-LRR genes often triggers immune responses^34^,^35^. An activated immune system is often accompanied by high expression of pathogenesis-related (*PR*) and defence-related genes^35^,^36^. Thus, we examined the transcripts of several pathogenesis-related (*PR*) genes in T51-1 and MH63 at the three-leaf stage. Although these seedlings were free from pathogens, the results showed that the expression of *OsPR1a*, *OsPR4a* and *OsPAD4* in T51-1 were up-regulated compared to MH63 (Fig. 1c); *OsPR2* was unchanged; and *OsPR5*, *OsPR8*, and *OsPR10a* decreased. These data indicate that *OsLSR* up-regulation activates the expression of some disease response genes, and the immune system in T51-1 is activated at a low level. The metabolic investment of a plant for a constitutively activated immune system consequently results in a high fitness cost and yield penalty^37^,^38^. Thus, the weakness of T51-1 can be attributed to its activated immune system.

### Transient expression of *OsLSR* triggers hypersensitive response liked cell death in tobacco leaf

**Figure 2:**
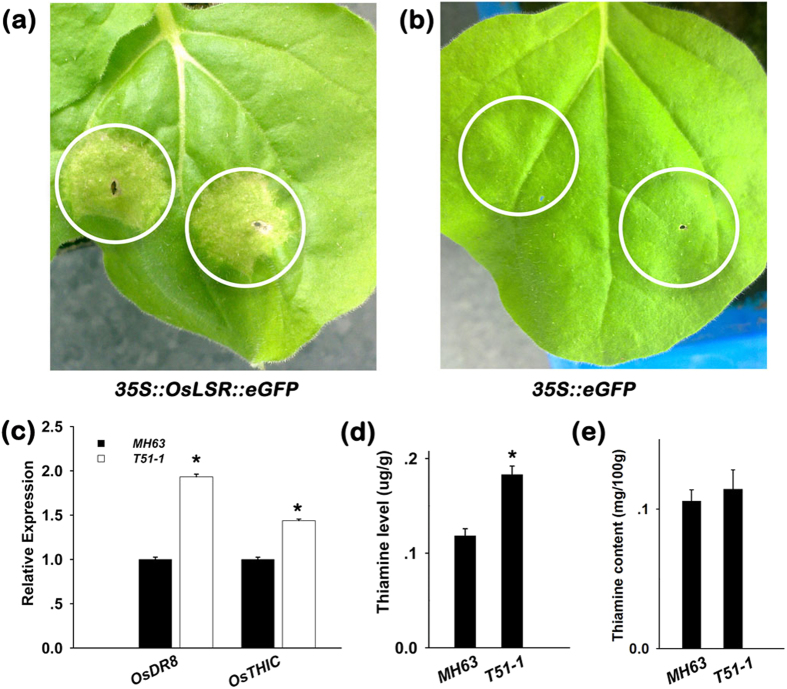
*OsLSR* induces HR-like cell death in tobacco leaf and activation of thiamine biosynthesis in rice. (**a,b**) *OsLSR* transient expression assays. HR-like cell death appears in the infiltration area of *OsLSR::eGFP* (**a**), eGFP (**b**) was used as a control. (**c**) Expression of rice genes homologous to plant thiamine biosynthesis *OsDR8*, *OsTHIC* in T51-1 and MH63. (**d**) Thiamine level in MH63 and T51-1 plants. (**e**) Thiamine level in brown rice of T51-1 and MH63. For the expression analysis, *OsUBQ1* was used as the internal control. Values are means ± SE (n = 3), *P < 0.05(ANOVA) comparing with T51-1 and MH63.

Transient overexpression of plant NB-LRR commonly leads to a hypersensitive response, which results in programmed cell death of infiltrated cells in tobacco leaf. Thus, we transiently expressed *OsLSR::eGFP* under control of the *35* *S* promoter in tobacco *Nicotiana benthamiana*. The construct triggered localized cell death in tobacco leaf (Fig. 2a–b), which was accompanied by H_2_O_2_ production and *NtPR1* expression in the infiltration area (Supplementary Fig. S1). This result demonstrated that transient overexpression of *OsLSR* can trigger the plant immune response and programmed cell death.

### Up-regulation of thiamine biosynthesis genes in T51-1

Plant NB-LRR genes activate the plant immune system through various pathways, including SA (salicylic acid), JA (jasmonic acid), etc. However, a RT-qPCR analysis showed that the SA and JA pathway-related genes were down regulated in T51-1 compared with MH63 (Supplementary Fig. S1). This result suggests that the upregulation of *OsLSR* did not activate JA or SA pathway. This was consistent with the results showing that some JA- and SA-responsive *PR* genes were not increased in T51-1(Fig. 1c).

A previous study showed that the basal expression of *OsPR1a* and *OsPR4a* is related to *OsDR8* because RNAi of *OsDR8* leads to the down regulation of *OsPR1a* and *OsPR4a*^19^. *OsDR8* encodes an enzyme involved in the biosynthesis of the thiazole precursor of thiamine (VB1). Thus, the transcript levels of *OsDR8* and another rice gene *OsTHIC* (*Os03g0679700*) homologous to the *Arabidopsis AtTHIC*, which is critical in thiamine biosynthesis and regulation, were analysed in T51-1 and MH63. The results showed that their transcript levels were all increased in T51-1 (Fig. 2c). Then, we examined the thiamine content in the leaf tissue of both T51-1 and MH63. Correlating with the expression analysis, T51-1 showed a 0.5-fold increase in the thiamine level compared with that of MH63 (Fig. 2d). However, both T51-1 and MH63 accumulated a similar level of thiamine in their brown rice (*P* = 0.578) (Fig. 2e). Thus, our results showed that the slight upregulation of *OsLSR* expression activated the de novo synthesis of VB1 in rice leaf.

### Overexpression of *OsLSR::eGFP* results in autoactivation of immune response and accumulation of thiamine through up regulation of *OsDR8*

To further understand the functional characteristics of *OsLSR*, we created *OsLSR* overexpressing (*OsLSR*^*OE*^) lines in *Nipponbare* under the control of the *ACTIN1* promoter. These lines showed high expression of *OsLSR* (Supplementary Fig. S2). A phenotypic evaluation showed that overexpressing *OsLSR* in rice results in an extremely low seed setting rate (Supplementary Table S1). Moreover, the *OsLSR*^*OE*^ lines showed a severe weakness compared with the control *Nipponbare* (Supplementary Fig. S2). Seeds carrying the *OsLSR*^*OE*^ construct failed to germinate and form seedlings (Supplementary Fig. S2).

**Figure 3:**
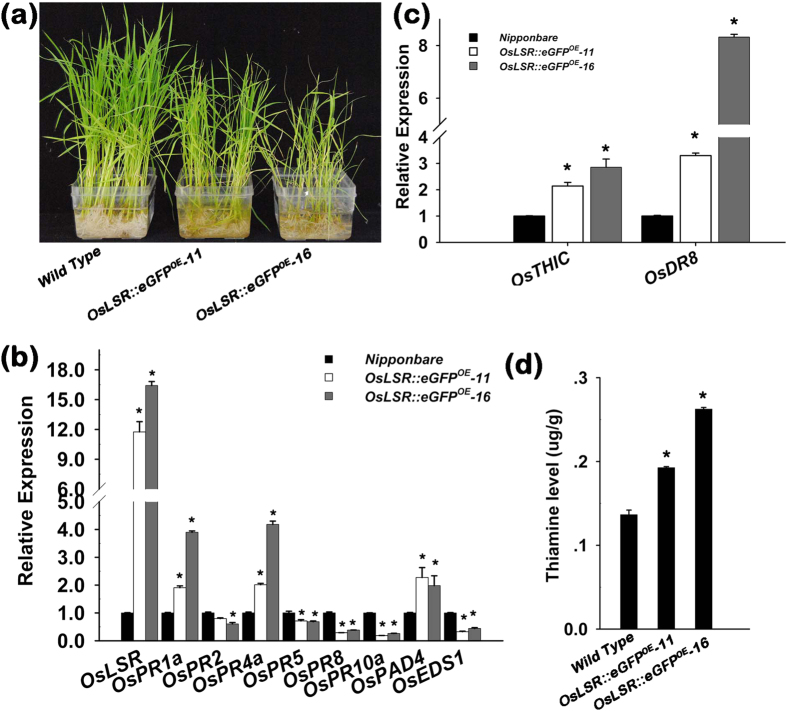
Molecular characterization of *OsLSR::eGFP*^*OE*^ plants. (**a**) Growth retardation of *OsLSR::eGFP*^*OE*^*-11,-16* (T3) plants. (**b**) Expression level of *OsLSR* and the immune response related genes in *OsLSR::eGFP*^*OE*^ lines. (**c**) Transcript level of thiamine biosynthesis genes in *OsLSR::eGFP*^*OE*^ lines. (**d**) Thiamine level examined in the leaf tissue of *Nipponbare* (wild type) and *OsLSR::eGFP*^*OE*^ plants. For the expression analysis, *OsUBQ1* was used as the internal control. Values are means ± SE (n = 3). *P < 0.05 (ANOVA) comparing WT plants and two *OsLSR::eGFP*^*OE*^ lines.

Thus we further overexpressed an eGFP fusion *OsLSR::eGFP* in *Nipponbare* under the control of the 35 S promoter. Transgenic rice overexpressing *OsLSR::eGFP* showed minor weakness compared with the wildtype at three leaf stage (Fig. 3a). Relative quantitative PCR showed that the two *OsLSR::eGFP*^*OE*^*-11,-16* lines of T3 generation highly expressed the foreign fusion, and resistance marker genes showed a similar expression pattern as that in T51-1, *OsPR1a*, *OsPR4a* and *OsPAD4* were up regulated while the rest were down-regulated (Fig. 3b), indicating the function of OsLSR was unlikely affected by the fused eGFP. Further transcript screening of thiamine synthesis-related genes revealed that the expressions of *OsTHIC* and *OsDR8* were increased particularly for *OsDR8* in these lines compared with wildtype *Nipponbare* (Fig. 3c). These changes correlated with the expression level of *OsLSR::eGFP* (Fig. 3b). In parallel, the thiamine contents in these two *OsLSR::eGFP*^*OE*^ lines increased by 0.5~1-fold compared with the wild type *Nipponbare* (Fig. 3d). These results further verified that the up regulation of *OsLSR* results in autoactivation of immune response and accumulation of thiamine. It was well documented that maintaining an activated immune system is resource-consuming, and a high level of thiamine results in increased activity of TPP-requiring enzymes to result in enhanced carbohydrate oxidation^37^,^39^. Thus, these plants suffered a great fitness cost and showed weakness, especially in growth and seed setting (Supplementary Table S1).

### Overexpression of *OsLSR1-437* activates *PR* genes expression in rice

**Figure 4:**
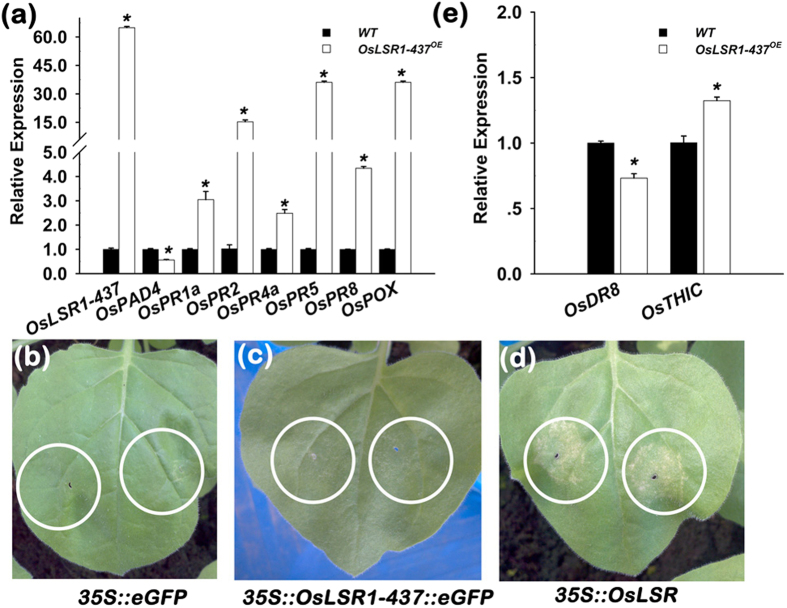
Molecular analyses and transient expression assay of *OsLSR1-437*^*OE*^ plants. (**a**) Expression analysis of immune response genes in *OsLSR1-437*^*OE*^ plants. (**b–d**) Transient expression assay of *OsLSR1-437::eGFP* in tobacco leaf. Free eGFP was used as a negative control (**b**), *OsLSR1-437::eGFP* (**c**), (**d**) *35* *S::OsLSR* was used as a positive control. (**e**) Expression analysis of two thiamine biosynthesis enzyme-encoding genes in *OsLSR1-437*^*OE*^ plants. For the expression analysis, *OsUBQ1* was used as the internal control. Values are means ± SE (n = 3). *P < 0.05 (ANOVA) comparing WT plants and *OsLSR1-437*^*OE*^ plants.

Several studies have shown that ectopic expression of certain parts of the NB-LRR protein can trigger an immune response^40^. Based on this consideration, we overexpressed the N-terminal 437aa of *OsLSR* under the *ACTIN1* promoter in *Nipponbare* (*OsLSR1-437*^*OE*^) and eventually obtained one transgenic line. T3 seedlings positive for OsLSR1-437 at the three-leaf stage were used for RT-qPCR analysis. The results showed that the fragment was efficiently expressed, whereas the immune response genes showed a different pattern from *OsLSR::eGEP*^*OE*^ lines. All the detected *PR* genes were up regulated compared with wild type (Fig. 4a) and at a level much higher than that in *OsLSR::eGFP*^*OE*^ and T51-1, but *OsPAD4* was decreased. These results suggest that the N-terminal portion of OsLSR (the CC plus NB domain) can successfully trigger transcriptional reprogramming when overexpressed in rice. However, when *OsLSR1-437::eGFP* under the control of the 35 S promoter was transiently expressed in tobacco leaf, the construct failed to trigger HR-like cell death comparing with *35* *S::OsLSR* (Fig. 4b–d). Furthermore, an expression analysis of thiamine biosynthesis genes showed that only *OsTHIC* increased slightly, while *OsDR8* was down regulated (Fig. 4e). These results suggest that the N-terminal portion of OsLSR (the CC plus NB domain) partly lost *OsLSR*’s signalling property when overexpressed in rice or transient expressed in tobacco leaf. LRR domain was reported also critical in signalling function for some NB-LRR proteins^41^. These results indicate that remove of the LRR domain disables *OsLSR* in cell death and thiamine biosynthesis signalling, while the N-terminus could conducts transcriptional reprogramming when overexpressed. These results also imply that *OsLSR*-triggered transcriptional reprogramming and thiamine biosynthesis are two independent processes.

### Down regulation of *OsLSR* by RNA interference also leads to autoactivation of immune response and accumulation of thiamine by promoting *OsTHIC*

**Figure 5:**
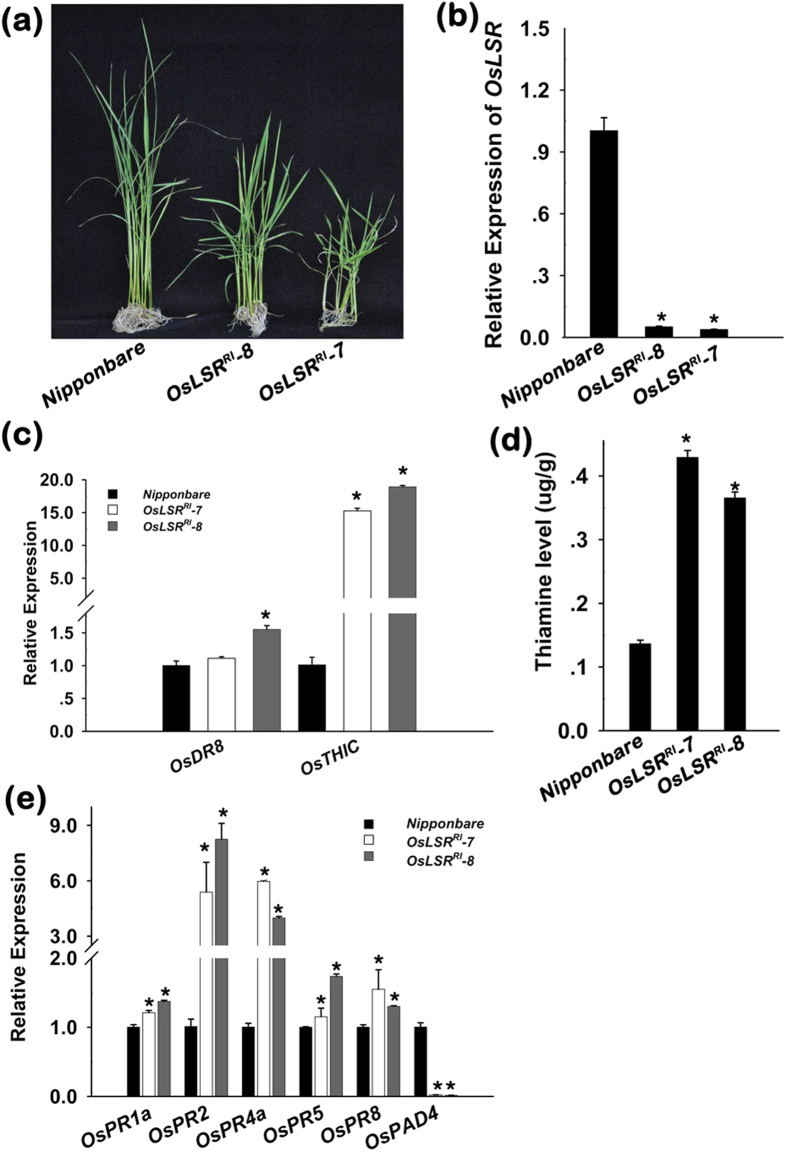
RNA interference of *OsLSR* and its impacts on rice growth and thiamine biosynthesis. (**a**) Weakness of *OsLSR*^*RI*^ plants (right and middle) and wild type *Nipponbare* (left). (**b**) Expression of *OsLSR* in *Nipponbare* and two typical *OsLSR*^*RI*^ lines. (**c**) Transcripts of thiamine biosynthesis genes in *OsLSR*^*RI*^ plants compared with wild type *Nipponbare*. (**d**) Thiamine level in *Nipponbare* and two *OsLSR*^*RI*^ independent lines. (**e**) Expression of immune response genes in *OsLSR*^*RI*^ lines compared with *Nipponbare*. For the expression analysis, *OsUBQ1* was used as the internal control. Values are means ± SE (n = 3). *P < 0.05 (ANOVA) comparing *Nipponbare* plants and two RNAi lines.

To investigate the effect of knocking out *OsLSR*, the expression of *OsLSR* in *Nipponbare* was blocked by RNA interference (*OsLSR*^*RI*^). *OsLSR*^*RI*^ lines showed not only delayed growth compared to wild type *Nipponbare*, but even more severe growth retardant than *OsLSR::eGFP*^*OE*^ plants (Fig. 5a), and they also displayed a low seed setting phenotype (Supplementary Table S1). The expression levels of *OsLSR* in two representative lines, *OsLSR*^*RI*^*-7* and *OsLSR*^*RI*^*-8*, of the T3 generation at seedling stage were analysed. The results showed that the lines had 95% and 96% inhibition, respectively (Fig. 5b). However, an expression profile analysis revealed a different pattern of thiamine biosynthesis genes from wild type. The down regulation of *OsLSR* resulted in increased *OsDR8*, *OsTHIC*; in particular, *OsTHIC* was considerably increased (Fig. 5c). Like *OsLSR::eGFP*^*OE*^ plants the two *OsLSR*^*RI*^ lines showed increased thiamine content compared to wild type plants (Fig. 5d), and the level was even higher than that in *OsLSR::eGFP*^*OE*^ plants. Transcript analyse of *PR* genes showed they were all increased by a certain amount, indicating silence of *OsLSR* also results in autoactivation of immune response (Fig. 5e). Interestingly, *OsPAD4* was down regulated in the *OsLSR*^*RI*^ lines (Fig. 5e), indicating that *OsLSR* plays a signalling role upstream of *OsPAD4*, and positively regulates its expression.

### Gene expression changes in *OsLSR* transgenic plants

**Figure 6:**
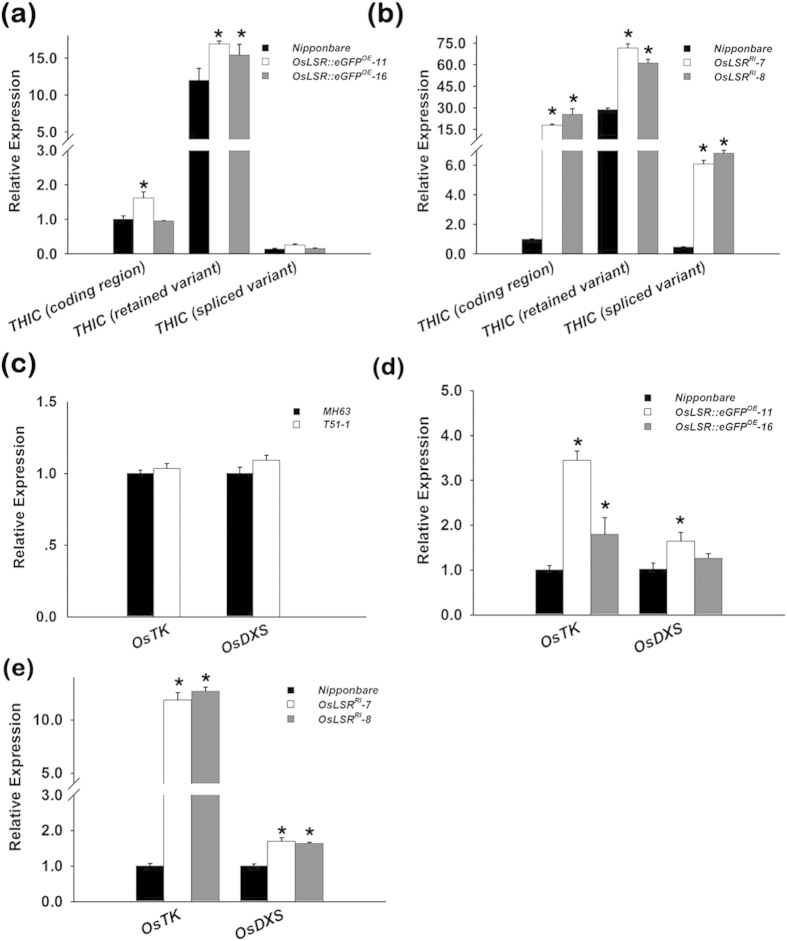
Gene expression analysis of *OsLSR* transgenic plants. Expression level of *OsTHIC* alternative transcripts in *OsLSR::eGFP*^*OE*^ (**a**) and *OsLSR*^*RI*^ (**b**) plants. Gene expressions of rice transketolase (TK) and deoyxylulose-5-phosphate synthase (DXS) in T51-1 and MH63 (**c**), *OsLSR::eGFP*^*OE*^ (**d**), and *OsLSR*^*RI*^ (**e**) lines. For the expression analysis, *OsUBQ1* was used as the internal control. Values are means ± SE (n = 3). *P < 0.05 (ANOVA) comparing *Nipponbare* plants and two RNAi lines.

The biosynthesis of thiamine is tightly and negatively regulated by TPP through a riboswitch on the *THIC* mRNA^27^. A high level of TPP in cells leads to an increased level of the unstable mRNA form of *THIC*, in which the second intron in the 3′UTR is spliced out; when the TPP level is low, this intron is retained, resulting in enhanced stability of the mRNA and increased translation of the THIC protein. An expression analysis of these types of *OsTHIC* mRNA in *OsLSR::eGFP*^*OE*^ and *OsLSR*^*RI*^ plants showed an increase in the intron-retained type (Fig. 6a,b). In the *OsLSR*^*RI*^ lines, all three types of *OsTHIC* mRNA were increased. Taken together, these results suggest that the expression pattern of *OsTHIC* alternative transcripts in *OsLSR* transgenic plants is consistent with a high thiamine level.

The plant Calvin Benson (C3) cycle provides two major precursors for thiamine biosynthesis, G3P (Glyceraldehyde 3-phosphate) and R5-P (Ribose 5-phosphate). Two C3 cycle enzymes, transketolase (TK) and deoyxylulose-5-phosphate synthase (DXS) are critical for their metabolism^22^,^42^. We analysed the expression of these enzymes in the transgenic plants. Results showed *OsTK* (*Os01g0931400*) and *OsDSX* (*Os05g0408900*) were increased in both *OsLSR::eGFP*^*OE*^ and *OsLSR*^*RI*^ lines, however, the two genes were not significant up regulated in T51-1 (Fig. 6c–e). This result indicates that *OsLSR* might affect thiamine biosynthesis at an upstream step, and the accurate regulation of *OsLSR* is important for plant growth and development because a disturbance in its expression results in metabolic chaos.

### Two fragments, OsLSR1–178 and OsLSR464–546, are targeted to the chloroplast

Plant thiamine is biosynthesized in the chloroplast, whereas most cloned NB-LRR proteins are distributed in the nucleus or cytoplasm. To better understand the function of OsLSR and its relationship with thiamine biosynthesis, we constructed a C-terminal fusion with eGFP to study the distribution of OsLSR in the plant cell. However, we were unable to detect an eGFP signal after transient expression in tobacco leaves. In addition, we were still unable to detect the eGFP signal after steady overexpression in rice plants. One possibility is that the fusion protein triggers cell death, tobacco cells were breakdown before the fusion accumulated to a visible level, transgenic rice cells with high fusion protein level were aborted during early stage of *Agrobacterium* mediated transformation, and only these compromised transgenic lines were survived.

**Figure 7:**
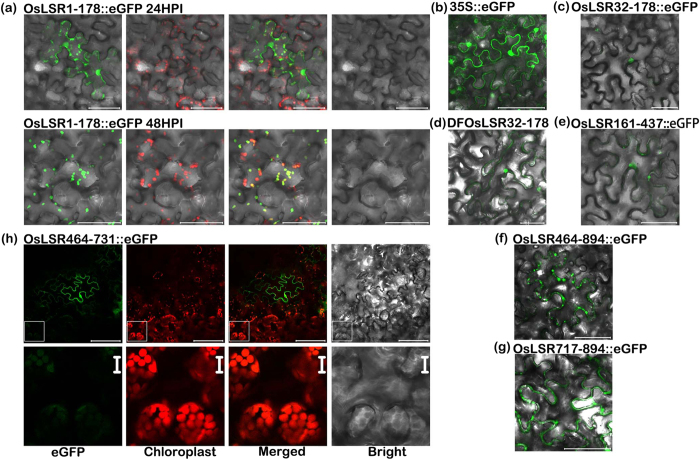
Subcellular localization of constructs carrying truncated OsLSR domains. (**a**) Subcellular distribution pattern of OsLSR1-178::eGFP showing a nuclear, chloroplast and cytoplasmic distribution pattern at 24 h after infiltration (upper panel). At 48 h, only chloroplast localization was observed (bottom panel). (**b**) Subcellular localization of eGFP (CK). (**c**) Subcellular localization pattern of OsLSR32–178::eGFP and DFOsLSR32–178::eGFP (**d**). Both constructs showed a nuclear-preferred distribution. (**e**) Subcellular distribution of OsLSR161-437::eGFP. Subcellular localization pattern of OsLSR464–894::eGFP (**f**) and OsLSR717–894::eGFP (**g**). Both constructs showed cytoplasmic distribution with non-uniform, punctate staining. (**h**) Subcellular localization of a truncated OsLSR LRR fragment, OsLSR464–731. Two sets of images are shown: a large-scale view (top panel) and a detailed view of the area indicated by the square (bottom panel). The fusion construct showed cytoplasmic distribution in a cell with strong signal (shown in upper panel), while localization to the chloroplast was observed in a cell with a weak signal (bottom panel). Scale bar, 50 μm. For OsLSR464–731::eGFP, the scale bar is 50 μm in the upper panel and 10 μm in the bottom panels.

Several studies on the subcellular distribution of NB-LRRs have shown that its individual domains play a role and contribute to the distribution of the intact protein^43^,^44^. Thus, we examined the distribution of individual domains of OsLSR to speculate about its subcellular location. We constructed a series of eGFP fusions that each contained a different truncation of OsLSR (Supplementary Fig. S3). All of the fusions could be visualized. The fusions containing OsLSR1–31 and OsLSR1–89 exhibited a nucleo-cytoplasmic distribution similar to control 35 S::eGFP (Supplementary Fig. S3). The fusion containing OsLSR1–178, which includes the CC domain plus 50aa, showed a distinct distribution pattern. At 24HPI (hour post infiltration), the eGFP signal was widely distributed in chloroplasts, the nucleus, and the cytoplasm. As time progressed to 48HPI, the fusion was observable only in chloroplasts (Fig. 7a).

A fusion containing OsLSR32–178 was constructed to test whether a chloroplast signal was still produced. This fusion did not produce a signal in chloroplasts and tended to be located in the nucleus with very little signal in the cytoplasm (Fig. 7c). To determine whether the nuclear accumulation of OsLSR32–178::eGFP resulted from active nuclear import via a nuclear targeting signal or passive import through diffusion, we added another eGFP to its N-terminus and named the construct DFLSR32–178::eGFP. This construct still displayed nuclear localization, although the additional eGFP increased its retention in the cytoplasm in tobacco leaf cells (Fig. 7d). These results indicated that OsLSR has a positive NLS in the OsLSR32–178 region, and full-length OsLSR1–178 is necessary to target the protein to the chloroplast.

The construct containing OsLSR161–437, which corresponds to the ATPase domain, displayed nuclear localization with very little fluorescence in the cytoplasm (Fig. 7e). The fusion containing OsLSR464–894, which includes the putative LRR domain or its truncated fragment OsLSR717–894, localized in the cytoplasm (Fig. 7f,g).

Unexpectedly, constructs carrying truncated sequences from the N-terminus of OsLSR464–894, such as OsLSR464–546, OsLSR464–731, and OsLSR464–867, showed a distribution pattern that was very different from either OsLSR464–894 or OsLSR717–894. In cells with strong signals, the fluorescence pattern clearly outlined the tobacco epidermis cells, indicating that the fusions were located in the cytoplasm (Fig. 7h, Supplementary Fig. S3).By contrast, in cells with weak signals, the eGFP fluorescence overlapped with chloroplasts, indicating that the fusions located to the chloroplasts (Fig. 7h, Supplementary Fig. S3). Thus, the original target of these constructs was the chloroplast; however, the constructs localized to the cytoplasm when they were transiently overexpressed. This pattern of subcellular distribution is similar to that of OsLSR1–178::eGFP. The difference between the patterns is that the former three fusions no longer remained in the chloroplast during transient overexpression. These results revealed a subtle relationship between OsLSR truncations and the chloroplast.

## Discussion

Our results demonstrate that up- or down- regulation of *OsLSR* results in activated immune system of transgenic rice. These plants showed a weakness and yellowish phenotype, they constitutively express *PR* genes compared with wild type. Moreover these transgenic plants had a higher thiamine level in cells.

*OsLSR* can trigger immune response. This characteristic of *OsLSR* was evidenced by several experiments. First, in *OsLSR* up regulation lines, including T51-1 and *OsLSR::eGFP*^*OE*^, increases in defence-related and *PR* genes were observed. An increase in a set of *PR* and defence-related gene transcripts is a reliable molecular marker to indicate whether the plant immune system is activated^35^,^36^. Second, transiently overexpressed *OsLSR* or its *eGFP* fusion in tobacco leaf resulted in HR-like cell death. Timely programmed cell death of infected cells in the *R* gene-mediated defence response is an effective strategy for plants to restrict pathogens from spreading to other healthy cells^45^. Third, strong expression of *PR* genes was observed in rice ectopically expressing the CC plus NB domains of *OsLSR*, suggesting that overexpressed *OsLSR1–437* triggers rice transcripts reprogram which is common in an immune response. The N-terminal portion of NB-LRR was thought to be involved in downstream signal transduction. Previous reports indicated that overexpression of the N-terminal portion of some NB-LRR proteins results in auto-activation of the plant immune system^40^. Thus, *OsLSR* clearly acts as an activator of the plant immune system and can activate the plant defence response after activation or up-regulation; its N-terminal domain is involved in downstream defence signalling.

Different subsets of defence signalling cascades can be activated by *R* genes mediated resistance^46^,^47^. Our results showed that the expression of *OsPAD4* was associated with the transcriptional level of *OsLSR*. In *OsLSR* up regulation lines, *OsPAD4* expression was increased, while in *OsLSR*^*RI*^ lines, expression was decreased. These results indicate that *OsLSR* functions upstream of *OsPAD4* and positively regulates its expression. Furthermore, *OsLSR* may function as a signalling factor downstream of immune receptors because it is required for proper expression of *OsPAD4*. Interestingly, strong expression of *PR* genes but with the down regulation of *OsPAD4* was observed in rice plants ectopically expressing *OsLSR1–437*, suggest OsLSR1–437 lost the native specificity of OsLSR in downstream immune signalling when overexpressed, which was also observed in several cases exploring NB-LRR protein function^40^. *OsPAD4* encodes a putative triacylglycerol lipase, and positively regulates rice defence responses to bacterial *Xanthomonas oryzae pv. Oryzae* (*Xoo*) in JA pathway^48^. In Arabidopsis, pathogen-induced *PR1a* expression is dependent on *AtPAD4*^49^, this observation aligns with the increased expression of *OsPR1a* in *OsLSR* up regulated lines. However, different from *OsPAD4*-mediated bacterial blight resistance is JA dependent, in T51-1 SA and JA synthetic genes were inhibited, indicating *OsLSR* activated immune signalling does not activate JA and SA. This might because plants possess multi mechanisms to activate immune system besides SA and JA, and some time one activated immune signal interact antagonism to another. Antagonism interaction was found between SA and JA when plants facing biotrophic or necrotrophic pathogen invasion in Arabidopsis. Suppression of JA and SA synthesis related genes with enhanced resistance to *Xoo* infection was also reported in rice overexpressing an IAA-amino acid synthetase gene *GH3–8*^50^,^51^. More efforts are needed to elucidate details of the immune signalling pathway in *OsLSR* up- and down- regulated lines, and it is of interesting to systematically evaluate the disease resistance property of *OsLSR* transgenic rice plants in future study.

Another interesting thing in the present study is we found that up-regulation or silencing of *OsLSR* result in accumulation of thiamine in rice. In T51-1, 0.8 fold increase of *OsLSR* results in 0.5 fold higher of thiamine level compared with MH63 in its leaf tissue. However, the thiamine level in its brown rice was increased not significantly. Similar results were also observed in the *Arabidopsis* plants overexpressing *AtTHIC*, which had three-fold increase of the thiamine level in their leaf tissue but only 20% increase in its seeds compared with wildtype plants^39^,^52^. These results indicated that simply up-regulation of thiamine synthetic genes in plants is insufficiently to cause considerable increase of the thiamine level in its storage organ. The interesting thing is that in *OsLSR::eGFP*^*OE*^*-11*, while the expression of *OsLSR*, *OsDR8* and *OsTHIC* was much higher than that in T51-1, the two lines had similar thiamine level. This may be because the two lines are of different genetic background, as the thiamine level in *OsLSR::eGFP*^*OE*^ lines with the same background is positively related with the expression of *OsLSR*, *OsTHIC* and *OsDR8* (Fig. 3).

The higher thiamine level in *OsLSR*^*RI*^ lines could be explained by the fact that in *OsLSR::eGFP*^*OE*^ lines, *OsDR8* increased more than *OsTHIC*; however, in *OsLSR*^*RI*^ plants, a strong increase in the *OsTHIC* intron-retained variant was observed compared to a minor increase in *OsDR8*. The *THIC* intron-retained variant is more effective than *TH1* in the induction of thiamine biosynthesis when it is overexpressed^23^,^39^. Further, *OsTK* and *OsDXS* which provides precursors for thiamine biosynthesis is more enhanced in *OsLSR*^*RI*^ lines than *OsLSR::eGFP*^*OE*^ lines. These results imply that the signal triggered for thiamine biosynthesis in these two lines is different. *OsLSR::eGFP*^*OE*^ plants tend to activate thiamine biosynthesis through thiazole moieties pathway, while *OsLSR*^*RI*^ plants mainly by activating the other pyrimidine moiety pathway. In addition, the above results also indicate that *OsLSR* might have a role in balancing the provision of both moieties of HMPP and HET-P used for thiamine biosynthesis. This balancing was found to be critical in *Chlamydomonas reinhardtii*, which guarantees that sufficient but not excess thiamine is available^34^,^53^.

Thiamine is synthesized in the chloroplast^22^,^42^. In the experiment, the data from the assay of eGFP-tagged OsLSR fragments showed that the chloroplast is the primary target of OsLSR1–178 and OsLSR428–546 after transient expression in tobacco leaf cells. And further experiments showed the two fragments had an interaction in yeast. Taking together, these results indicate that these two fragments may help OsLSR target to chloroplast and affects thiamine synthesis there.

In the present study, we have shown that both overexpression and RNA silencing of *OsLSR* results in weakness of the transgenic rice plants, including growth retardation, chlorosis and deceased seed setting. One explanation for the phenotype is their constitutively activated immune system, as these defects were common in plant mutants with an auto-activated immune system^54^,^55^,^56^. Maintaining an activated immune system is costly, and fitness-limiting resources and energy that are allocated to resistance are unavailable for fitness-relevant processes, such as growth, and cause penalties in reproduction^37^,^38^. These results indicate that NB-LRR genes should be tightly controlled to avoid unnecessary activation, which is harmful to plants per se.

An alternative explanation for the weakness of *OsLSR* transgenic plants is the elevated thiamine level in the plants. Thiamine and its active form TPP are an essential cofactor for enzymes involved in a number of critical metabolic processes, such as the production of acetyl-CoA, the tricarboxylic acid cycle (TCA), the pentose phosphate pathway (PPP), the C3 cycle, etc.^57^. Excess available TPP in a cell increased the activity of TPP-requiring enzymes, resulting in an increase in carbohydrate oxidation through TCA, PPP, and the accumulation of amino acids. This augmented flux through central metabolism that perturbs metabolic homeostasis results in chlorosis, growth retardation, etc.^39^,^52^. In this study, abnormal expression of *OsTK* and *OsDXS* which evolve in C3 cycle is observed indicating a metabolic chaos in these transgenic rice plants.

Our results presented here showed that both up-and down-regulation of *OsLSR* leads to autoactivation of rice immune system and accumulation of thiamine results in a weakness phenotype. Thus, there exists an unknown mechanism regulates *OsLSR* within a narrow range for the normal growth and development of rice plants. More efforts are needed to explore the function of *OsLSR* as its expression is closely related to thiamine level in cells and immunity which could be used for rice improvement.

## Materials and Methods

### Plant materials

*Oryza sativa* L. subsp. *Indica* line MH63 (wild type controls), T51-1 (MH63 background), the *japonica* line *Nipponbare* and transgenic plants or lines were grown in the field or greenhouse at Zijingang campus, Zhejiang University. The growth conditions in the greenhouse were set to a photoperiod of 12 h light at 28 °C/12 h dark at 25 °C, with a relative humidity of approximately 60%. *Nicotiana benthamiana* was grown in a growth chamber under a photoperiod of 12 h light at 28 °C/12 h dark at 22 °C, with a relative humidity of approximately 50%.

### Vector construction

Total RNA was isolated from the leaf tissue of *Nipponbare* plants using TRIzol reagent (Invitrogen), according to the manufacturer’s instruction. First-strand cDNA synthesis was conducted using the M-MLV first-strand synthesis system (Promega). To construct the overexpression vector, the complete coding sequence (CDS) of *OsLSR* was amplified using the gene-specific primers LSR^OE^-F and LSR^OE^-R (Supplementary Table S2) with XbaI and KpnI restriction sites, respectively. The amplified cDNA products were cloned into p1300-actin:NOS^58^ to produce p1300-actin::OsLSR::NOS, which was named pOELSR.

The RNAi vector was constructed using a 350 bp fragment corresponding to the 2806 bp to 3155 bp region of *Os10g0183000*; the fragment was amplified using the primers LSR^Ri^-F and LSR^Ri^-R. The product was digested with two pairs of restriction enzymes (*Bam*HI/*Kpn*I and *Spe*I/*Sac*I), and the products were cloned into pTCK303^59^ to produce the RNAi repression construct pRiLSR. The pOELSR and pRiLSR vectors were introduced into *Agrobacterium tumefaciens* strain EHA105, which was used to infect rice embryonic calli from Nipponbare to generate *OsLSR* overexpressing plants (*OsLSR*^OE^) and RNA interfering (*OsLSR*^RI^) plants, respectively.

To construct the subcellular localization vectors, the complete CDS or truncations of OsLSR were amplified using the primers listed in Supplementary Table S2; all forward and reverse primers contained XbaI and KpnI restriction sites, respectively. The PCR products were then inserted into p1300-35S::eGFP::NOS to generate the corresponding vectors.

### Quantification of thiamine content

The thiamine level in the leaf tissue of rice plants was determined using a fluorescent method^19^,^21^. In brief, ~2–4 g of rice leaf tissue was ground into a powder in liquid nitrogen. Thiamine was extracted in 0.1 M HCl, and thiamine was oxidized into thiochrome in a solution containing 0.1% kalium ferricyanide and 15% sodium hydroxide. The fluorescence emitted by the thiochrome was detected at 365 nm excitation and 435 nm emission wavelengths using an ELISA (Biotech Synergy H1).

### Transient expression assay in *Nicotiana benthamiana*

The vectors were transformed into *A.tumefaciens EH105* for transient expression. *A. tumefaciens* cells were grown in 5 ml LB with the appropriate antibiotics overnight. One hundred microliters of the overnight culture was used to inoculate 25 ml of fresh LB (with the same antibiotics as the overnight culture, plus 20 μM acetosyringone added after autoclaving and immediately before use) and grown to an OD600 of 1.0. The cells were then collected by centrifugation and resuspended in the induction medium (10 mM MES, pH 5.7, 10 mM MgCl_2_, 200 μM acetosyringone) to an OD600 of 0.4. After incubation at room temperature for 2–3 h, the *A. tumefaciens* cells were infiltrated into the abaxial surface of tobacco leaves using 1 ml needle syringes. eGFP signals were observed using a laser scanning confocal microscope (Leica TCS 710).

### Real-time quantitative RT-PCR

For real-time quantitative RT-PCR, leaf samples were harvested at the three-leaf stage; three biological repeats for each sample were used. Total RNA from the leaves was isolated using an RNA extraction kit (TRIzol reagent, Invitrogen). Approximately 1 μg of total RNA was reverse-transcribed using a PrimeScript^TM^ RT reagent kit with gDNA Eraser (TAKARA) in a final volume of 20 μl and then diluted to 100 μl. Quantitative RT-PCR was performed in a total volume of 20 μl, using 2 μl of the reverse-transcribed product above, 0.2 μM primers and 10 μl of Fast Start Essential DNA Green Master (Roche) on a Roche LC96. The amounts of transcript were estimated using the relative quantification method^60^ with *UBQ1* as internal standards for normalization. The primers used in this experiment are listed in Supplementary Table S3.

### Phenotype evaluation of transgenic rice

Seed setting rate of MH63, T51-1 was the mean value of ten independent plants. For each plant only productive tillerings are taken in consider. Seed setting for transgenic material: certain independent lines indicated in tables were evaluated. For each independent line, ten plants were evaluated, and the mean value of each transgenic line is further statistically analyzed to get standard error for each transgenic material.

## Additional Information

**Accession codes:** *OsLSR:* Os10g0183000, *OsDXS:* Os05g0408900, *OsTHIC*: Os03g0679700, *OsTK:* Os06g0133800.

**How to cite this article**: Wang, L. *et al*. Both overexpression and suppression of an *Oryza sativa* NB-LRR-like gene *OsLSR* result in autoactivation of immune response and thiamine accumulation. *Sci. Rep*. **6**, 24079; doi: 10.1038/srep24079 (2016).

## Supplementary Material

Supplementary Information
