# Supplementary material for: Both overexpression and suppression of an Oryza sativa NB-LRR-like gene OsLSR result in autoactivation of immune response and thiamine accumulation
Source: Sci Rep. 2016 Apr 7;6:24079. doi: 10.1038/srep24079 (PMC4823736; doi:10.1038/srep24079)
Supplement: Supplementary Information [file srep24079-s1.pdf]

Scientific reports supplementary information

Article title: Both overexpression and suppression of an *Oryza sativa* NB-LRR-like gene *OsLSR* result in autoactivation of immune response and thiamine accumulation

Authors: Wang Liangchao, Ye Xiufen, Liu Huachun, Liu Xuejiao, Huang Yuqing, Wei Chuchu, Liu Yujun, Tu Jumin*

The following supplementary information is available for this article:

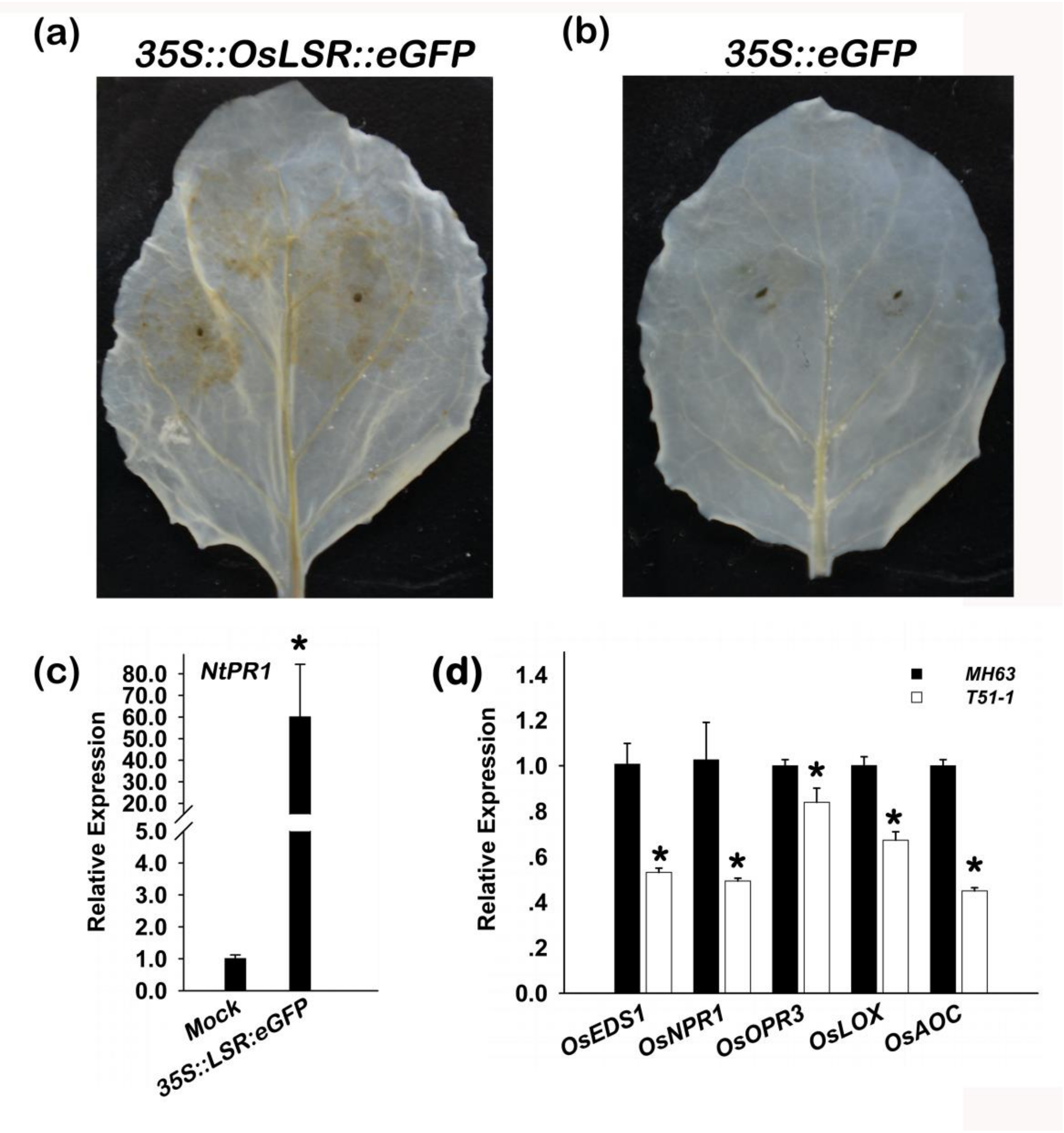

Figure. S1 Diaminobenzidine (DAB) staining of H_2O_2 and *NtPR1a* detection in tobacco leaf

transiently expressing *OsLSR*. (a-b) DAB staining of H_2O_2 following transient expression of *OsLSR* (a); transient expression of eGFP (b) does not induce any staining. (c) Relative expression of *NtPR1a* in infiltration area of tobacoo leaf transient expression of *OsLSR* or Mock. (d) Relative expression of SA (salicylic acid) and JA (jasmonic acid)-related pathway genes in MH63 and T51-1.Values are means ± SE (n =3). *P < 0.05 (ANOVA).

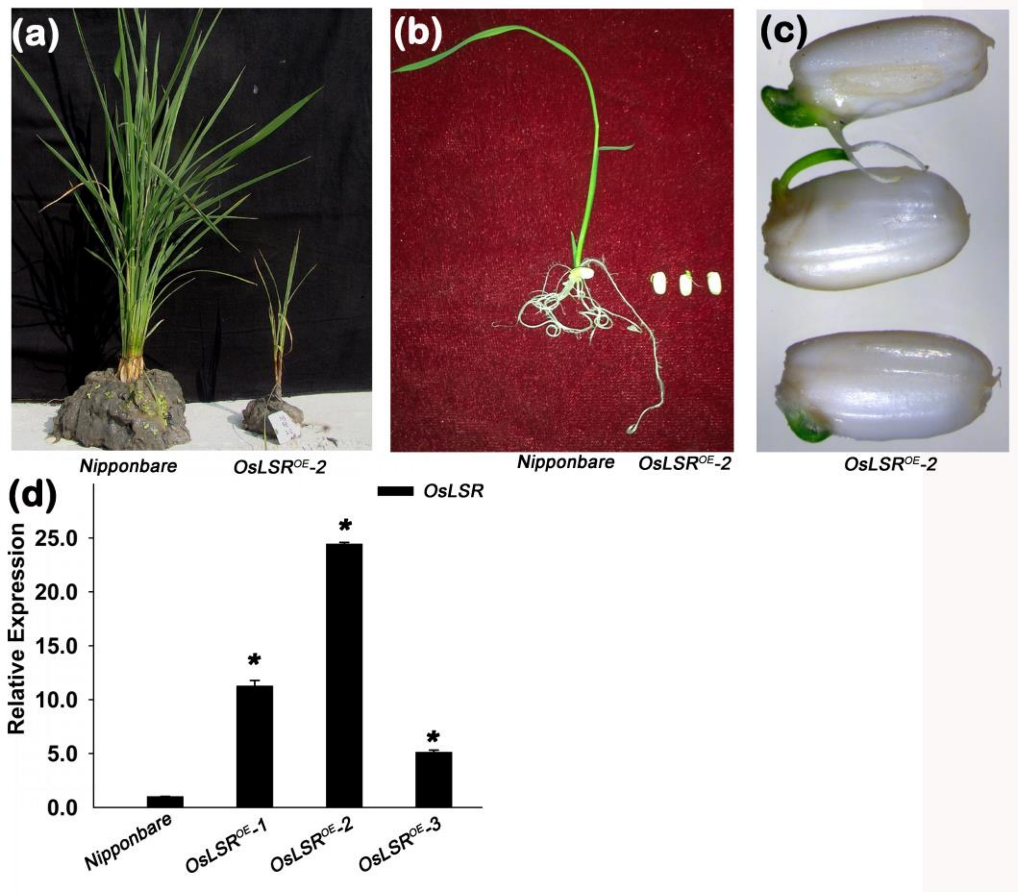

Figure. S2 Phenotype evaluation of *OsLSR*^{OE} lines**.** **(**a). Plant morphology of wild type and *OsLSR*^{OE}-2 during the tilling stage. (b) *OsLSR*^{OE}*-2* positive seeds failed to germinate. (c) Detailed view of *OsLSR*^{OE}*-2* seeds in (b). (d) Relative expression of *OsLSR* in wild type (WT) *Nipponbare* and three independent *OsLSR*^{OE} lines. Values are means ± SE (n =3). *P < 0.05 (ANOVA) comparing WT plants and *OsLSR*^{OE} lines.

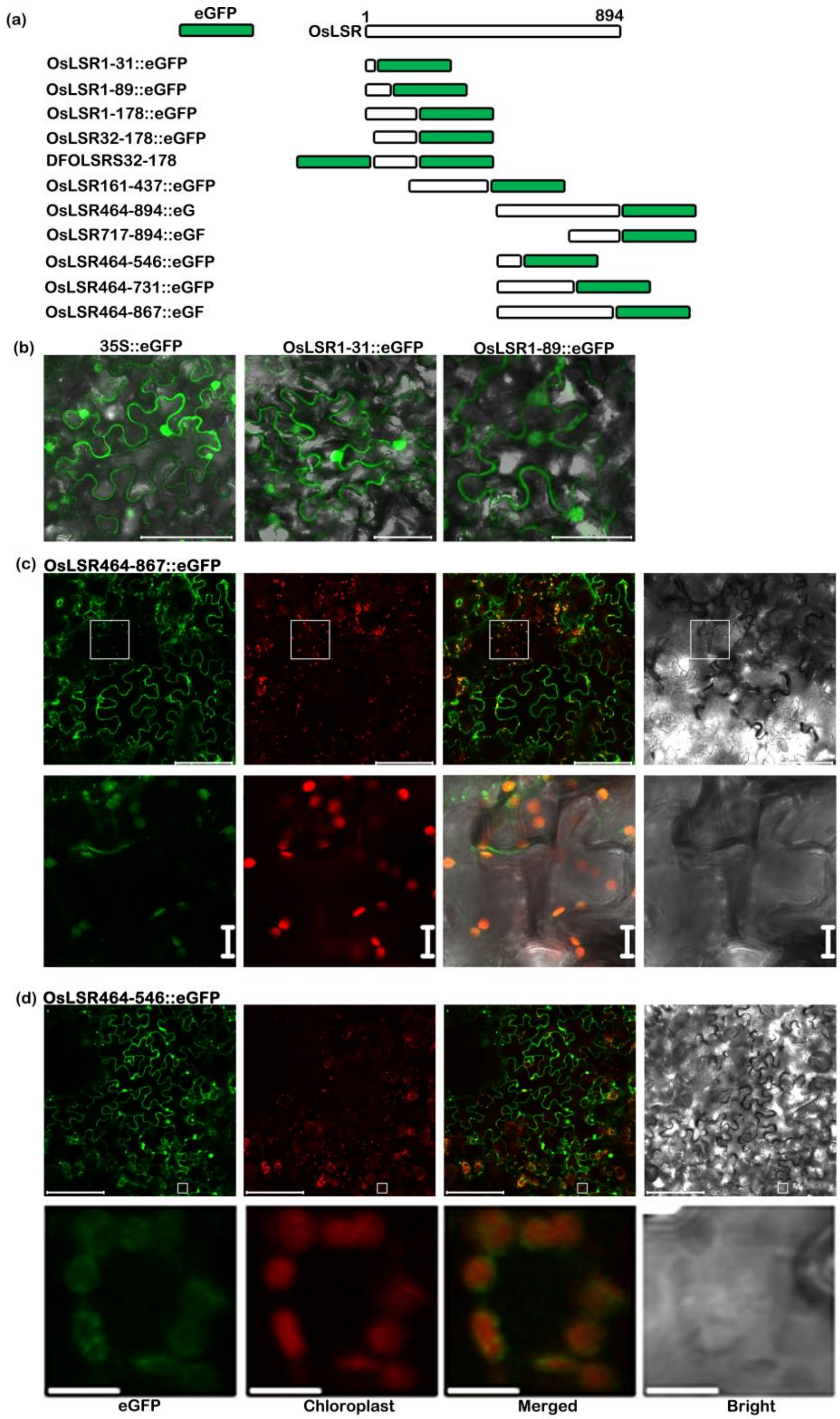

Figure. S3 Subcellular localization of constructs carrying truncated OsLSR domains. (a) Structure map of vectors with eGFP fusion containing OsLSR domain truncations. The length

of each truncated domain is drawn to scale. (b) Subcellular localization of eGFP (CK), OsLSR1-31::eGFP, and OsLSR1-89::eGFP. All show a nuclear and cytoplasmic pattern of distribution. (c-d) Subcellular distributions of the truncated LRR domain OsLSR464-867::eGFP (c), OsLSR464-546::eGFP (d). Scale bar in (b) is 50um, for (b-c) 50 µm in top panels, and 2 µm in bottom panels.

Table S1. Seed setting rate of rice MH63, T51-1, $OsLSR^{OE}$, $OsLSR::eGFP^{OE}$ and $OsLSR1\text{-}437^{OE}$. ± SE.

Material	Number of Independent line	Seed setting rate (%)
MH63	1	86.6 ±2.34
T51-1	1	75.2 ±2.45
Nipponbare	1	91.84 ±1.82
$OsLSR^{OE}$(T0)	10	11.11 ±7.86
$OsLSR::eGFP^{OE}$	5	16.14 ±5.25
$OsLSR1\text{-}437^{OE}$	1	6.86 ±1.56
$OsLSR^{RI}$	13	12.4 ± 12.05

Table S2. List of primers and corresponding sequences with introduced restriction (marked and underlined) sites used in vector construction.

Gene/domain	Primers	Sequences (5'-3')
**Construction of OsLSR overexpressing, RNA interference OsLSR::sGFP or its domain truncation::eGFP fusion vectors**		
OsLSR	LSR^{OE}-f	GGGGTCTAGAATGACAGCATCAGTAGTTTC
	LSR^{OE}-r	GGGGGAGCTCTCAAGTATCAGAAGTTTGTTCCTC
	LSR^{Ri}-f	GGGGGGTACCACTAGTatggcgtttctcgctgtt
	LSR^{Ri}-r	GGGGGGATCCGAGCTCttgcaaatgcaagtgatg
OsLSR::eGFP	LSR-eGFPf-*Xba*I	GGGGTCTAGAAACAATGACAGCATCAGTAGTTTC
	LSR-eGFPr-*Kpn*I	GGGGGGTACCAGTATCAGAAGTTTGTTCCTCGTCAAC
OsLSR1-31::eGFP	LSR-eGFPf-*Xba*I	GGGGTCTAGAAACAATGACAGCATCAGTAGTTTC
	LSR31-eGFPr-*Kpn*I	GGGGGGTACCTACTTGTCGAGGAACATCAC
LSR1-89::eGFP	LSR-eGFPf-*Xba*I	GGGGTCTAGAAACAATGACAGCATCAGTAGTTTC
	LSR89-eGFPr-*Kpn*I	GGGGGGTACCCTTTCTCCTCCTAAAAATGAAATG
LSR1-178::eGFP	LSR-eGFPf-*Xba*I	GGGGTCTAGAAACAATGACAGCATCAGTAGTTTC
	LSR178-eGFPr-*Kpn*I	GGGGGGTACCGTAACTCTTAATTTGTGCAA
LSR32-178::eGFP	LSR32-eGFPf-*Xba*I	GGGGTCTAGAAACAATGTCATCCATGAAAGCAGAACTG
	LSR178-eGFPr-*Kpn*I	GGGGGGTACCGTAACTCTTAATTTGTGCAA
LSR161-437::eGFP	LSR161-eGFPf-*Xba*I	GGGGTCTAGAAACAATGGGGCAAGGAATGGATAT
	LSR437-eGFPr-*Kpn*I	GGGGGGTACCGATAGGGTAGTCCTCGG
LSR464-894::eGFP	LSR464-eGFPf-*Xba*I	GGGGTCTAGAAACAATGGGGGCCTACAGATATGT
	LSR-eGFPr-*Kpn*I	GGGGGGTACCAGTATCAGAAGTTTGTTCCTCGTCAAC
LSR717-894::eGFP	LSR717-eGFPf-*Xba*I	GGGGTCTAGAAACAATGGAACATGAAGAATACAATTT
	LSR-eGFPr-*Kpn*I	GGGGGGTACCAGTATCAGAAGTTTGTTCCTCGTCAAC
LSR464-54:6::eGFP	LSR546-eGFPr-*Kpn*I	GGGGGGTACCTTTTCCCACTTCATTATCAAAGTAA
LSR464-731::eGFP	LSR731-eGFPr-*Kpn*I	GGGGGGTACCAACTTTGGATTCTAAGGCATCTACC
LSR464-867::eGFP	LSR867-eGFPr-*Kpn*I	GGGGGGTACCGAGCTGAGGCATGCAAATCA
**Construction of DFLSR32-178::eGFPfusion vector**		
OsLSR32-178	YFP-up-*Xba*I 2	GGGGTCTAGAAACAATGGTGAGCAAGGGCGAGGA
	YFP-down-*Spe*I 2	GGGGACTAGTCCCACTCGAGTCGACGCAGATC
**Construction of vectors used in Y2H**		
OsLSR1-178	LSR-Y2Hf-*Nde*I	GGGGCATATGACAGCATCAGTAGTTTC
	LSR178-Y2Hr-*Eco*RI	GGGGGAATTCTCAGTCAAGCAAGTAACTCTTAA
OsLSR161-437	LSR161-Y2Hf-*Nde*I	GGGGCATATGCAAGGAATGGATATAGTTGG
	LSR437-Y2Hr-*Eco*RI	GGGGGAATTCTCAGATAGGGTAGTCCTCGG
OsLSR428-894	LSR428-Y2Hf-*Nde*I	GGGGCATATGactgcatcctttcccgagg
	LRR894-Y2Hr-*Eco*RI	GGGGGAATTCTCAAGTATCAGAAGTTTGTTCC
OsLSR161-463	LSR161-Y2Hf-*Nde*I	GGGGCATATGCAAGGAATGGATATAGTTGG
	LSR463-Y2Hr-*Eco*RI	GGGGGAATTCaacttcttcttgtgtatatcc
OsLSR464-546	LSR464-Y2Hf-*Nde*I	GGGGCATATGGCCTACAGATATGT
	LSR546-BKr-*Eco*RI	GGGGGAATTCTTTTCCCACTTCATTATCAAAGTAA
OsLSR	LSR-Y2Hr-*Eco*RI	GGGGGAATTCATGACAGCATCAGTAGTTTC
	LSR-ADf-*Kpn*I	GGGGGGTACCTCAAGTATCAGAAGTTTGTTCCTC
	LSR-BKf-*Pst*I	GGGGCTGCAGTCAAGTATCAGAAGTTTGTTCCTC
OsLSR428-546	LSR546-BKr-*Eco*RI	GGGGGAATTCTTTTCCCACTTCATTATCAAAGTAA
OsLSR428-731	LSR731-BKr-*Eco*RI	GGGGGAATTCAACTTTGGATTCTAAGGCATCTACC
OsLSR428-867	LSR867-BKr-*Eco*RI	GGGGGAATTCGAGCTGAGGCATGCAAATCA

Table S3. List of primers corresponding sequences used in quantification of gene expression.

Gene	Primers	Sequences (5'-3')
**Tobacco**		
JN247448.1	NtPR1af	gtgcccaaaattctcaacaag
	NtPR1ar	ttctacacctacatctgcacgag
AJ421411.1	a tublinf	atggcttgctgccttatgtt
	a tublinr	cacagcagcattgacatcct
**Rice**		
OsLSR	OsLSR f	atgccgtgtcatgatttcag
	OsLSR r	gattattttgctgggaaaccta
Os07g0129200	PR1a f	TCGTATGCTATGCTACGTGTTT
	PR1a r	CACTAAGCAAATACGGCTGACA
Os01g0940700	PR2f	AAGATTGTTCTGAGAAGAGATCGATCGA
	PR2r	GCTACGCGAAAATAGGTCTGGTAAACTT
Os11g0592200	PR4af	GATGCCAACAAACCGTTGTC
	PR4ar	CGCAATTATTGTCGCACCTG
Os07g0418500	P450f	TTCTATAAGAGTTTGCCACTTCG
	P450r	CAAATACATTGTCTGAATAACATCAA
Os12g0628600	PR5 r	CAACAGCAACTACCAAGTCGTCTT
	PR5 f	CAAGGTGTCGTTTTATTCATCAACTTT
Os10g0416500	PR8 r	GTTCATCTGGTCAGCGGATAGC
	PR8 f	TCATAAGTATTATCACGACCGTTCGA
Os12g0555500	PR10a f	TGTGTGGCCAAGCTCAAGGT
	PR10a r	GACGAGGTAGTCCTCGATCA
Os03g0438100	aocf	tccccttcaccaacaagc
	aocr	gctgtagatggcctcgtagc
Os08g0508800	loxf	tctccctcgagctcgtca
	loxr	gtagctccgcacagttgct
Os08g0459600	opr3f	ggcatctttttctgccagtt
	opr3r	caccgtttggctggtatactt
Os11g0195500	pad4f	caggggttcttgaggctgt
	pad4r	aacactactgccttgcctctg
Os09g0361500	EDS1 r	acaagggctgatgactttcaa
	EDS1 f	cgcagatcatctccagctt
Os07g0677200	POX r	GCCATCATGGACGGTTCTGT
	POX f	TCTGGAGAAATTGCCGATAAGTTC
Os03g0234200	UBQ1f	CTGTCAACTGCCGCAAGAAG
	UBQ1r	GGCGAGTGACGCTCTAGTTC
Os05g0408900	DXS f	tcagcaagctgcagtcca
	DXS r	cctccgatttgcttcgtc
Os06g0133800	TK f	ctacgacgccgtcttgga
	TK r	tcgaagttctcgggatgg
Os03g0679700	THIC coding region r	ttgggcaaaagatgtgcag
	THIC coding region f	ttttcaggaattttatgcatcg
	DR8 r	tcagctcctcctcctctcg
	DR8 f	agcgaaggaggtcttgagg
Os03g0679700	THIC intron spliced r	gaaagctatacagctcgcaaataa
	THIC intron spliced f	acaacaatccaggccaagag
	THIC intron retained r	gaaagctatacagctcgcaaataa
	THIC intron retained f	tgagctaagcaacaccagctt
